# Ketoanalogues supplementation decreases dialysis and mortality risk in patients with anemic advanced chronic kidney disease

**DOI:** 10.1371/journal.pone.0176847

**Published:** 2017-05-05

**Authors:** Che-Hsiung Wu, Ya-Wen Yang, Szu-Chun Hung, Ko-Lin Kuo, Kwan-Dun Wu, Vin-Cent Wu, Tsung-Cheng Hsieh, for the National Taiwan University Study Group on Acute Renal Failure (NSARF)

**Affiliations:** 1 Division of Nephrology, Taipei Tzu Chi Hospital, Buddhist Tzu Chi Medical Foundation, Taipei, Taiwan; 2 Institute of Medical Sciences, Tzu Chi University, Hualien, Taiwan; 3 School of Medicine, Tzu Chi University, Hualien, Taiwan; 4 Division of General Surgery, Department of Surgery, National Taiwan University Hospital, Taipei, Taiwan; 5 Division of Nephrology, Department of Internal Medicine, National Taiwan University Hospital, Taipei, Taiwan; Hospital Universitario de la Princesa, SPAIN

## Abstract

**Background:**

The benefit of alpha-Ketoanalogues (KA) supplementation for chronic kidney disease (CKD) patients that followed low-protein diet (LPD) remains undetermined.

**Methods:**

We extracted longitudinal data for all CKD patients in the Taiwan National Health Insurance from January 1, 2000 through December 31, 2010. A total of 1483 patients with anemic advanced CKD treated with LPD, who started KA supplementation, were enrolled in this study. We analyzed the risks of end stage renal disease and all-cause mortality using Cox proportional hazard models with influential drugs as time-dependent variables.

**Results:**

A total of 1113 events of initiating long-term dialysis and 1228 events of the composite outcome of long-term dialysis or death occurred in patients with advanced CKD after a mean follow-up of 1.57 years. Data analysis suggests KA supplementation is associated with a lower risk for long-term dialysis and the composite outcome when daily dosage is more than 5.5 tablets. The beneficial effect was consistent in subgroup analysis, independent of age, sex, and comorbidities.

**Conclusions:**

Among advanced CKD patients that followed LPD, KA supplementation at an appropriate dosage may substantially reduce the risk of initiating long-term dialysis or of developing the composite outcome. KA supplementation represents an additional therapeutic strategy to slow the progression of CKD.

## Introduction

Dietary protein has long been thought to play a pivotal role in the progression of chronic kidney disease (CKD), and a low protein diet (LPD) is usually recommended to patients with CKD to ameliorate uremic symptoms and slow the progression of renal dysfunction[1]. Dietary protein restriction preserves renal function and improves survival in animals with varies glomerulopathies[2, 3]. Conversely, it was suggested that prolonged protein restriction preceding dialysis may induce protein malnutrition and thus confer a poor prognosis during dialysis[4]. The prevalence of protein-energy wasting in early to moderate CKD is 20–25% and increases as CKD progresses[5]. Alpha-Ketoanalogues (KA) of essential amino acids, converted into essential amino acids in the body via transamination, could improve nutritional deficiencies caused by protein-restricted diets in CKD patients. In the 1980s, Mitch and colleague found that using KA to supplement a LPD slowed or halted the progression of renal insufficiency in CKD patients[6]. LPD supplemented with KA alleviated the decrease in glomerular filtration rate (GFR) and maintained body mass index [7]. Other studies further suggest that KA per se may be renoprotective [8, 9]. However, a meta-analysis indicated that KA supplementation has an insignificant effect on preserving renal function [10, 11]. The reasons for the discrepancies between the results of studies conducted to evaluate LPD are of particular interest. Comparison of their designs reveals great heterogeneity due to short follow-up periods, late commencing supplementation, or small sample size. Therefore, data are still lacking regarding whether KA supplementation exerts an effect on decreasing risk for long-term dialysis or all-cause mortality in advanced CKD patients. As maintenance dialysis is generally initiated when uremic symptoms begin, its necessity may be deferred by LPD-KA.

In Taiwan, with an extremely high incidence and prevalence of end-stage renal disease (ESRD) [12, 13], patients start dialysis with very low residual renal function (average estimated GFR < 5.0 mL/min/1.73 m^2^) [14]. This clinical situation allows for a risk-benefit analysis of treatment in patients with advanced CKD using national claim databases[15]. Taking advantage of the Taiwan National Health Insurance (NHI) research database, we designed a nationwide, population-based cohort study to explore the association between KA supplementation and risk for long-term dialysis or all-cause mortality in advanced CKD patients. We also conducted a dose-response analysis regarding KA supplementation and outcome.

## Methods

### Data sources

The Taiwan National Health Insurance (NHI) is a nationwide insurance program that covers outpatient visits, hospital admissions, prescriptions, intervention procedures, and disease profiles for over 99% of the population in Taiwan (23.12 million in 2009). The NHI database is one of the largest and most comprehensive databases in the world, and offers research data for various studies on diagnoses, medication use, and hospitalizations [16, 17].

### Patient selection

Our cohort study used the longitudinal database created by the Taiwan National Health Research Institute (NHRI) by extracting original NHI data for all patients aged ≧ 20 years who were diagnosed with CKD and received ESA treatment in the NHI in the period from January 1, 2000 through December 31, 2010. Based on the reimbursement regulations of the NHI, ESA can only be prescribed in anemic advanced CKD patients with a hematocrit level of ≦ 28% and a serum creatinine level of > 6 mg/dL with the aim of achieving a hematocrit level of 33–36%. To ensure a sufficient duration of dialysis-free follow-up, we only enrolled patients not yet dialyzed (identified by procedure codes) from 6 months before to 90 days after the first ESA prescription.

**Fig 1:**
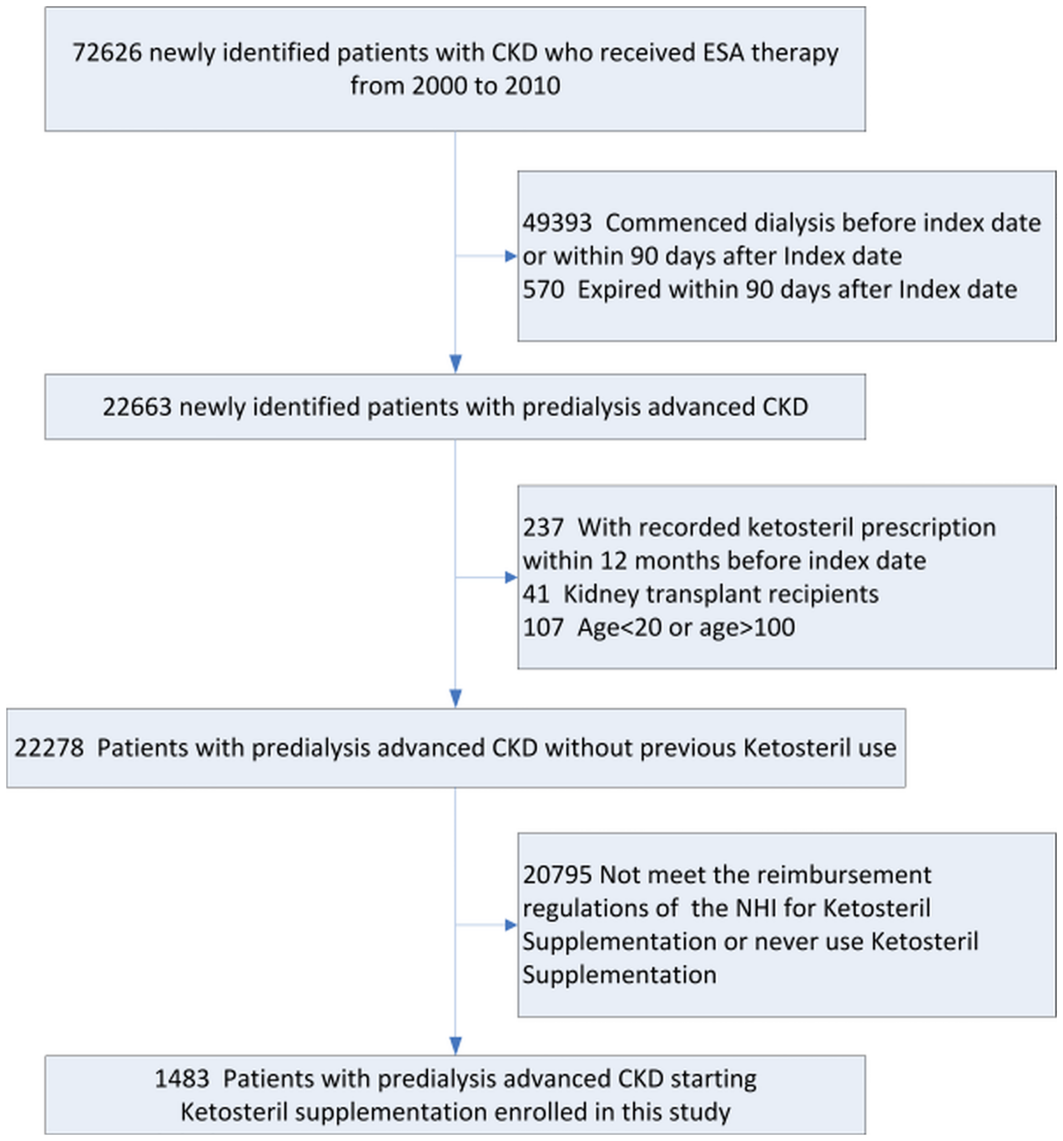
Flow diagram of study subject selection. (Abbreviations: CKD, chronic kidney disease; ESA, erythropoiesis stimulating agent; NHI, National Health Insurance).

According to NHI reimbursement regulations, patients in Taiwan with CKD who were on an LPD with serum creatinine levels above 6 mg/dL for three consecutive months could also receive low dose KA supplementation (Ketosteril^®^, Fresenius Kabi, Bad Homburg, Germany, Table A in S1 File) without copayment, with a maximum total dose of six tablets daily. The KA doses the patients were on were judged and determined by their attending physicians, typically one or two tables twice a day. Patient should be inspected at least once every two months; if serum creatinine level decrease to 5 mg/dL or less, or the compliance, evaluated by the dietitian to LPD was poor, KA supplementation should be discontinued immediately. Advanced CKD is defined as the presence of CKD coding and concomitant reimbursement of ESA prescriptions. The rate of ESA use was 85% in 2012 for patients with advanced CKD who had not yet commenced dialysis [18]. The first date of ESA prescription was defined as the index date. Patients were excluded if they had a KA prescription within the past 12 months before the index date, if they commenced dialysis before the index date, received dialysis or died within 90 days after the index date, or if they received a renal transplant. Ultimately, 1483 patients with predialysis stage 5 CKD starting KA supplementation were enrolled in this study (Fig 1).

### KA cumulated dose

The dose of KA was measured based on the cumulative dose in the 180 days preceding the 30^th^ day before outcomes. We used a long observation period to consider the lag effects of the drugs. In order to reduce potential confounding effects due to indication, we did not count medications prescribed within 30 days before the outcome event. In time-varying model, we could choose a selected fix- time period to calculate the drug dose. The time period is different from the Cox regression applied time varying model. The KA continuation period is a certain period of time during which the patient is with treatment of KA, and the countermanded period is a certain period of time during which the patient is without treatment of KA. The NHI claims data regarding medications are reliable because they were constructed on the basis of NHI procedures and drug codes that were tied to the NHI reimbursement system with auditing (Table B in S1 File).

### Multidisciplinary nutritional education

Since 2004, nearly all Taiwanese patients with a principal diagnosis of CKD have received a multidisciplinary care (MDC) program, thus reducing the probability of misdiagnosis [19–21]. The medical system in Taiwan has established a unique protocol to standardize and regulate pre-ESRD MDC, with all the medical expenditures covered by Taiwan NHI. For standardizing purposes, the management goals and education measures are stratified according to different stages of CKD and in compliance with the NKF K/DOQI guidelines. This program regulates that every patient diagnosed with CKD should receive specialist (including renal dietitian) consultations. The interventions evaluated vary considerably in terms of protein restriction (protein intake ≤0.8 g/kg/day). According to the reimbursement policy, patients with CKD stage 3 or 4 are followed in nephrology clinics every 3 months, while patients with CKD stage 5 are followed-up at least every month[19]. Nutritional compliance with dietary protein intake was measured by using diet record and analyzed using standard food composition tables. This Taiwanese nationwide CKD prevention program, including LPD and ESA use, was shown to be effective in reducing the incidence of ESRD, mortality, and medical costs [19–21].

### Research variables

We used codes from the International Classification of Disease, 9^th^ revision (ICD-9) to define diseases. The NHI data is generally reliable, because the National Health Insurance Administration routinely audits claims data to prevent fraud in the NHI program [22–25].

The baseline comorbidities, including CKD, were identified from at least three outpatient visits or one inpatient claim within one year preceding the first ESA prescription. This identification method has been well validated with good predictive power [16, 24, 26]. The Charlson comorbidity index was calculated by weighting baseline comorbidities[27] (Table C in S1 File). We also identified prescriptions that could be attributed to long-term dialysis or all-cause mortality risk, including angiotensin-converting enzyme inhibitor (ACEI), angiotensin II receptor blocker (ARB), and diuretics, within 30 days before the events.

### Outcome measurement

The observation period started from the index date to the date commencing long-term dialysis, to death, or to December 31, 2010, whichever occurred first. The primary endpoint was defined as long-term dialysis, the secondary endpoint was the composite outcome defined as long-term dialysis or all-cause mortality. In Taiwan, patients who continue dialysis for more than 90 days all apply a catastrophic illness registration card, which ensures the accuracy of our definition of dialysis continuation. The verification of ESRD on chronic dialysis includes a detailed exclusion of acute kidney injury; supported medical records, examination reports, and imaging studies with a comprehensive review by nephrology specialists are required [28].

### Data used for validation

The advanced CKD patients in Taiwan should be on LPD for three consecutive months before receiving KA supplementation without copayment. We validated the nutritional compliance with dietary protein intake by analyzing data of patients with CKD followed up in Taiwan Consortium for Acute Kidney Injury and Renal Diseases (CAKs) [29]. The purpose of CAKs is to build a platform on which we share the clinical data and exchange state-of-the-art research information. Up to June 2016, 21 hospitals along with the National Health Research Institute and Harvard statistics company have joined the Consortium. Through uniting Taiwan as a whole, CAKs aims to actively take part in the international kidney disease clinical trials and to pioneer in the scientific and bio-medical devices [30]. This study including the data from Taipei Tzu Chi hospital, and National Taiwan University Hospital and its 2 affiliated hospitals (National Taiwan University Hospital Yun-Lin branch, southern Taiwan, and National Taiwan University Hospital Hsin-Chu Branch, central Taiwan) during January 1, 2010 to August 31, 2015 attributed from CAKs.

### Statistical analysis

Continuous variables are described as mean ± SD; discrete variables are presented as counts and percentages. We used the R software, version 2.8.1 (Free Software Foundation, Inc., Boston, MA, U.S.A.). A two-sided p-value < 0.05 was considered statistically significant. KA supplementation is more often prescribed to patients whose renal function is expected by the physician to deteriorate more rapidly. Approaches using statistical methods to enroll those “unexposed” groups do not overcome this kind of initial selection bias. Therefore, we do not construct a control group using statistics matching. Instead, we used a Cox proportional hazards model with time-varying covariates to account for the influences of medications on the risks of long-term dialysis and the composite outcome in our investigation. Self- matching of cases eliminates the threat of control-selection bias and increases efficiency.

Time varying factors, including using KA, cumulated dose of KA, and ACEI/ARB as time-dependent covariate in the model, ensured that patients were at risk only when they have used them. The model allowed for moving patients from one exposure group to countermanding period. In the repeated analysis, person days of follow-up were correctly classified as untreated until the intended treatment definition of “each immortal time of use” was met, and as treated thereafter[31].

To determine threshold values of KA dosage for favorable renal outcome chances, we specified a multilevel discrete-time event history analysis using the logistic regression method incorporating patient-specific random effects, and adopted a generalized additive model (GAM) with splines regarding KA dosage. This approach permits adjustments for possible nonlinear effects of continuous variables [32, 33]. To show the effect of KA dosage on risk of initiating long-term dialysis, we plotted a function curve with values of the logs of odds ratios. The curve was centered to have an average of zero over the range of the data with approximate point-wise 95% CIs. After determining dosage threshold values, we categorized KA dosage levels accordingly, and incorporated the non-linear effect of KA dosage into the specification of another alternative Cox regression model to further examine the non-linear effect of KA dosage on outcome and avoid the healthy survivor bias[34]. Subgroup Cox regression analyses were further performed to examine the effects of age, sex, diabetics, hypertension, and nephrotic syndrome.

### Sensitivity analysis

To assess the reliability of our findings, additional analysis was conducted. We restricted the analysis to patients receiving ESA therapy at 2 or more consecutive ambulatory care visits to exclude acute exacerbation of chronic renal failure with transient creatinine levels of greater than 6mg/dL.

### Ethical considerations

Informed consent was originally obtained by the NHRI, and since patients were anonymous in the present study, informed consent was not required. Also, since the identification numbers of all individuals in the NHRI databases were encrypted to protect the privacy of the individuals, this study was exempt from a full ethical review by the institutional review board of National Taiwan University Hospital.

## Results

### Patient characteristics

A total of 1483 eligible patients with anemic advanced CKD who received KA therapy between January 1, 2000 and December 31, 2010 were enrolled in our study. Of the KA users (mean age 62.0±12.9 years), 44.5% (n = 660) were male, 26.5% (n = 393) had diabetes mellitus (DM), 59.7% (n = 885) had hypertension (HTN), and 4% (n = 59) had nephrotic syndrome. Table 1 summarizes the demographic and clinical characteristics of the enrollees.

**Table 1:** Clinical characteristics of KA users.

Characteristic	n = 1483
**Male Gender (%)**	660(44.5%)
**Age (year)**	62.04±12.91
**Baseline Comorbidities**	
Charlson comorbidity index	2.27±1.4
Myocardial infarction	17(1.1%)
Congestive heart failure	70(4.7%)
Peripheral vascular disease	5(0.3%)
Cerebrovascular disease	38(2.6%)
Dementia	8(0.5%)
Chronic obstructive pulmonary disease	65(4.4%)
Rheumatologic disease	14(0.9%)
Peptic Ulcer	183(12.3%)
Hemiplegia	3(0.2%)
Solid Tumor	64(4.3%)
Diabetes Mellitus	393(26.5%)
Moderate or severe liver disease	56(3.8%)
Hypertension	885(59.7%)
Stroke	38(2.6%)
Nephrotic syndrome	59(4%)
**Time-Dependent Variables**	
Drug use within 30 days before event	
Angiotensin-converting enzyme inhibitor	406 (27.4%)
Angiotensin II receptor blocker	802 (54.1%)
Diuretics	1046 (70.5%)

### Renoprotective effects of KA in patients with predialysis advanced CKD

A total of 1113 events of initiating long-term dialysis occurred in patients with predialysis advanced CKD during the mean follow-up period of 1.57 years. Table 2 lists risk factors for ESRD development among enrollees. The risk for long term dialysis was lower during the KA continuation period as compared to the countermanded period, with an HR of 0.52 (95% CI, 0.46–0.60; p < 0.001). Patients who had DM (HR 1.86; 95% CI 1.62–2.13; p < 0.001), HTN (HR 1.33; 95% CI 1.18–1.51; p < 0.001), and who were older than 60 years (HR 1.20; 95% CI 1.06–1.35; p = 0.003) had a higher risk of initiating long-term dialysis. The final Cox proportional hazards model with time-varying covariates had a good validity (C-index 0.8).

**Table 2:** Risk factors for chronic dialysis among patients with advanced chronic kidney disease.

Variables	Hazard Ratio (95% confidence interval)	p value
**Diabetes mellitus**	1.86 (1.62–2.13)	< 0.001
**Hypertension**	1.33 (1.18–1.51)	< 0.001
**Age, >60 vs. ≦60 years old**	1.20 (1.06–1.35)	0.003
**Daily KA dosage (expressed as tablets)** ^b^ **larger than 5.5 vs. Unsuitable dose**	0.64 (0.42–0.98)	0.0385
**ACEI use**	0.74 (0.58–0.94)	< 0.001
**ARB use**	0.76 (0.65–0.89)	< 0.001
**KA use**	0.52 (0.46–0.60)	< 0.001

^b^KA prescribed at less than 5.5 tablets daily is considered an unsuitable dose

Factors used in the Cox model: all covariates listed in Table 1

**Abbreviations:** ACEI, angiotensin-converting enzyme inhibitor; ARB, angiotensin II receptor antagonist; KA, Ketoanalog

### Dose-response analysis regarding KA supplementation and risk for long-term dialysis

**Fig 2:**
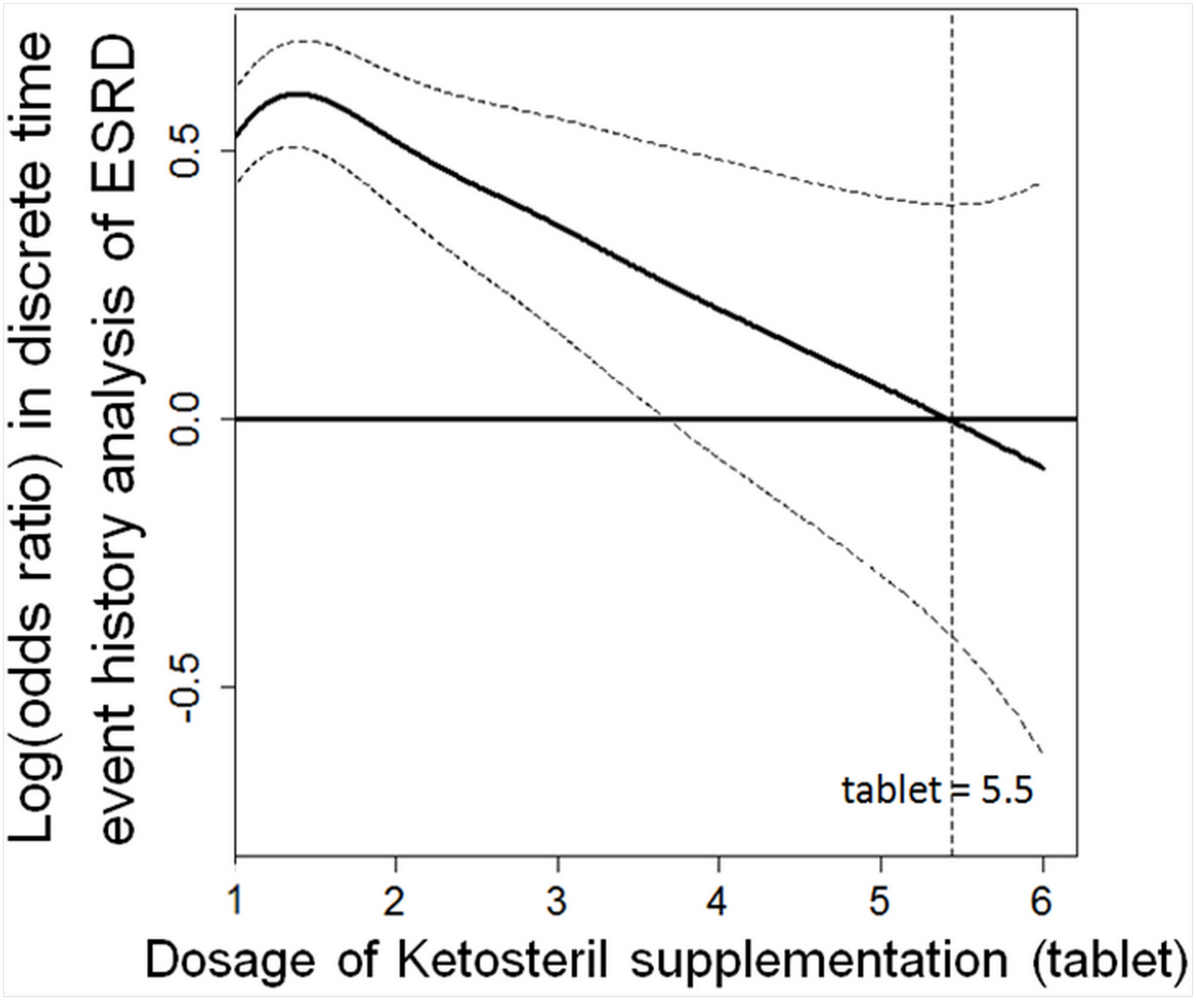
The function curve with values of the logs of odds ratios from the GAM with splines regarding KA supplementation for our multilevel discrete-time event history analysis of risk of commencing long-term dialysis among advanced chronic kidney disease patients. The curve was centered to have an average of zero over the range of the data. The dashed lines indicated approximated point-wise 95% CIs. (Abbreviations: CI, confidence interval; ESRD, end stage renal disease; GAM, generalized additive model; KA, Ketoanalogue).

Our generalized additive model (GAM) indicates the threshold value for the dosage of KA regarding long-term dialysis risk (Fig 2). We therefore defined less than 5.5 tablets daily of KA as an unsuitable dose. The HR of more than 5.5 tablets daily of KA relative to the unsuitable dose period was 0.65 (p = 0.049) (Table 2). This suggests that adequate use of KA may lower the risk of commencing long-term dialysis. In particular, more than 5.5 tablets of KA appeared to reduce the need for long term dialysis.

### Composite outcome after KA supplementation in patients with predialysis advanced CKD

A total of 1228 events of the composite outcome occurred in patients with predialysis advanced CKD. The risk for long term composite outcome was lower during the KA continuation period compared to the countermanded period (HR 0.43, 95% CI 0.38–0.49; p < 0.001). This protective effect was independent of the effects of DM (HR1.53; 95% CI 1.34–1.74; p < 0.001), HTN (HR 1.26; 95% CI 1.11–1.41; p < 0.001), and use of diuretic (HR 2.13; 95% CI 1.88–2.41; p < 0.001). Data analysis using the Cox regression model incorporating the non-linear effect of KA dosage on the composite outcome risk shows that the HR of a daily dosage of more than 5.5 tablets relative to the unsuitable dose period was 0.61 (p = 0.0225) (Table 3). For the composite outcome, our Cox proportional hazards model with time-varying covariates also had a good validity (C-index 0.81; Table 3). Again, it still showed an accumulated dose-dependent effect of KA on improving the composite outcome after daily use of more than 5.5 tablets. In line with our main findings, this suggests that adequate supplementation of KA may reduce the composite outcome risk.

**Table 3:** Risk factors for the composite outcome among patients with advanced chronic kidney disease.

Variables	Hazard Ratio (95% confidence interval)	p value
**Diabetes mellitus**	1.53 (1.34–1.74)	<0.001
**Hypertension**	1.26 (1.11–1.41)	<0.001
**Diuretic use**	2.13 (1.88–2.41)	<0.001
**KA use**	0.43 (0.38–0.49)	<0.001
**Daily KA dosage (expressed as tablets)** ^b^ **larger than 5.5 vs. Unsuitable dose**	0.61 (0.40–0.93)	0.0225

^b^KA prescribed at less than 5.5 tablets daily is considered an unsuitable dose

Factors used in the Cox model: all covariates listed in Table 1

**Abbreviations:** KA, Ketoanalog

### Comparison of the effect of KA supplementation on the risk of long-term dialysis and composite outcome under a framework of subgroup analysis

**Fig 3:**
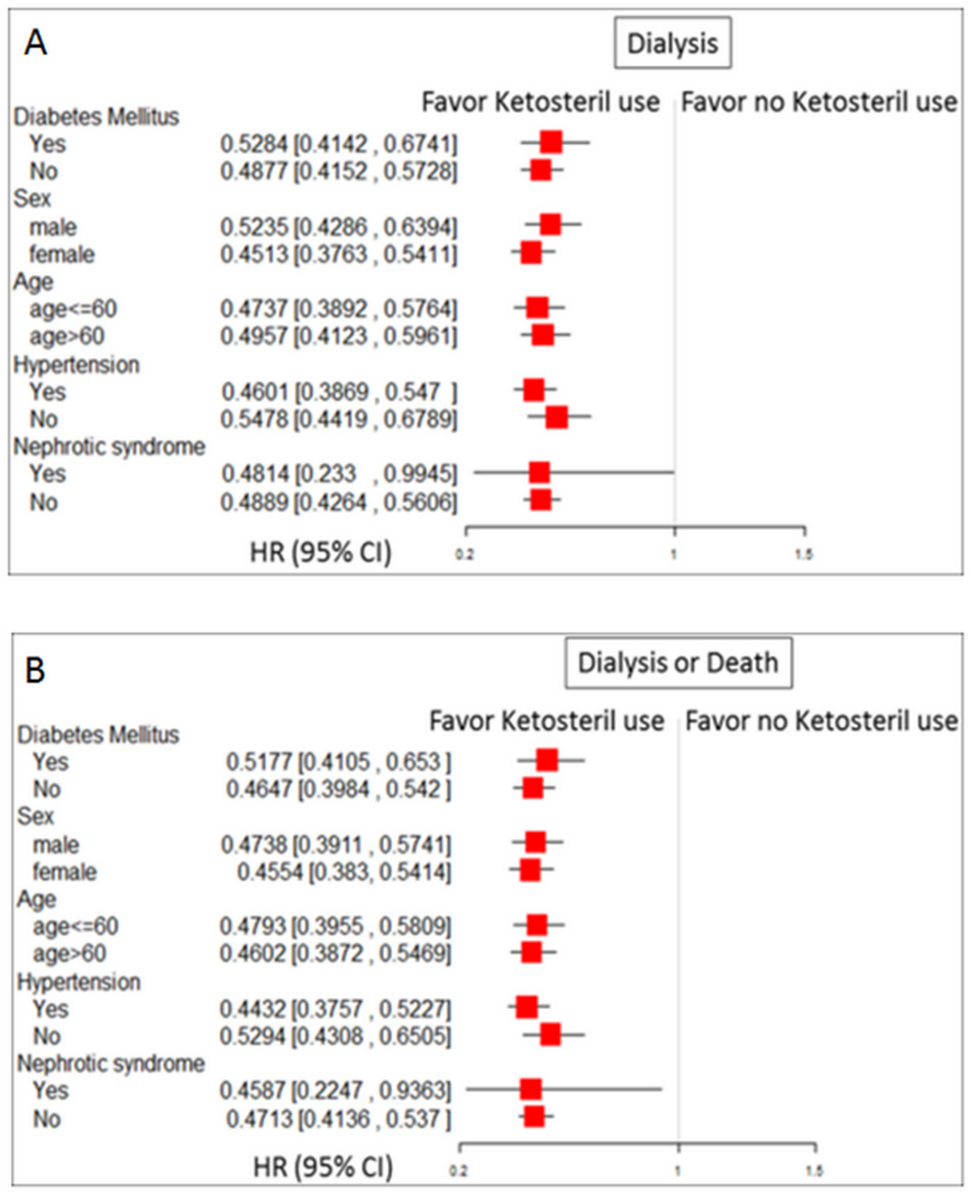
HRs of study outcomes among patients with advanced CKD, and subgroup analysis with respect to premorbid risk further adjusted for age and gender. (A) Long-term dialysis and (B) long-term dialysis or all-cause mortality. (Abbreviations: CI, confidence interval; CKD, chronic kidney disease, HR, hazard ratio)

To investigate the consistency in the beneficial effect of KA supplementation among different groups in advanced CKD patients, we conducted subgroup analysis. We found that KA supplementation was consistently associated with a much lower risk of long-term dialysis and composite outcome across broad varieties of patient groups with respect to baseline comorbidity as DM, HTN and nephrotic syndrome (Fig 3). In sensitivity analysis, we found that the estimated effect of KA supplementation was similar when we restricted analysis to patients receiving ESA therapy persistently (Tables D, E in S1 File).

### Characteristics of patient group validated by database attributed from CAKs

From patients with CKD followed up in CAKs from Taipei Tzu Chi hospital, and National Taiwan University Hospital and its 2 affiliated hospitals (National Taiwan University Hospital Yun-Lin branch, southern Taiwan, and National Taiwan University Hospital Hsin-Chu Branch, central Taiwan) during January 1, 2010 to August 31, 2015, we identified 655 patients with advanced CKD with serum creatinine more than 6 mg/dL. Sampled 62 patients received KA supplementation. The renal dietitians assessed the food intake of these patients using measuring bowls and food models. For calculating the amount of dietary protein per day, 3 day recall method was adopted. Renal dieticians calculated the amount of dietary protein per day using the Taiwan Food Composition database provided by the Taiwanese government. After 3 months of multidisciplinary nutritional education (before receiving KA supplement), the mean amount of dietary protein per day of KA user was 0.69 ± 0.05 g/kg per day.

The diagnosis of DM and HTN in NHI claims data were well validated [35]. Since NHI reimbursement regulation is strict, patients who did not change their diet to meet their bodies’ lower protein needs as nutritionist’s suggestion could not be candidate for KA treatment. Advanced CKD patients who have good adherence to LPD appeared to be a selected group of patients, and therefore the clinical characteristic of these patients were different from that in other reports. We validated the prevalence of DM and HTN by analyzing data attributed from CAKs as mentioned above. Among 62 patients with advanced CKD on LPD received KA supplementation, 18 patients had a diagnosis of DM (29.0%) and 41 had HTN (66.1%). This result was consistent with our data that the prevalence of DM and HTN in these selected groups of patients were lower than previous reports.

We also validated the decline in estimated glomerular filtration rate (eGFR) in advanced CKD patients (serum creatinine higher than 6 mg/dL) with and without KA supplementation. The decline in eGFR [using Modification of Diet in Renal Disease (MDRD) equation] after 3 months of multidisciplinary nutritional education in KA users and nonusers were 2.17 ± 3.19 mL/min/1.73m2 and 1.16 ± 1.52 mL/min/1.73m2, respectively (p<0.0001). KA supplementation seemed to be highly selected in real world practice. Therefore, we only enrolled patient who ever received KA to avoid indication bias.

## Discussion

Our results, derived from a large population cohort, provide evidence that KA supplementation during LPD may reduce the risk of commencing long-term dialysis or of developing the composite outcome in patients with advanced CKD, especially when used at an appropriate dosage. After a mean follow-up period of 1.57 years, KA supplementation with LPD decreased the risk of initiating long-term dialysis by 46%, and reduced the composite outcome risk in patients with advanced CKD by 51%. These benefits remained significant after subgroup analysis with respect to baseline comorbidity.

### KA ameliorates the risk of long-term dialysis

LPD shows the benefits of slowing kidney deterioration. However, it exposes patients to the risk of protein malnutrition [8, 36]. Potential strategies to avoid protein-energy wasting include supplementing the LPD with essential nutrients or supplements that are specifically designed for CKD patients[5].

Studies on animals with CKD showed that LPD supplemented with KA is more effective than an LPD alone in protecting remnant kidneys from progressive injury [37]. Compared with the LPD group, long term co-administration of an LPD and KA in CKD patients decreased the level of plasma asymmetric dimethylarginine and delayed the progression of renal failure [38]. The declining slopes of GFR during the LPD and KA period were significantly lower than those during the period of LPD alone [11].

Especially in older patients, dialysis may be associated with increased mortality risk and healthcare cost as compared with conservative care[39]. Renal replacement therapy can safely be deferred beyond 1 year by very LPD (0.3 g/kg) in advanced CKD patients[40]. Similarly, as shown in an Italian study, dietary protein restriction and KA supplementation could postpone the initiation of dialysis in elderly patients with CKD for about 1 year without increasing the risk of either death or hospitalization [41]. In the Hungarian KA Cohort Study, an LPD supplemented with KA could reduce the CKD progression rate.

Safely postponed initiation of dialysis could, on average, correspond to 21,180 euro/patient in the first, 6500 euro/patient in the second, and 682 euro/patient in the third year of treatment, with a significant net benefit in favor of dietary protein restriction with KA supplementation [42]. Postponing dialysis therapy should be cost saving because the incidence of stage 5 CKD grows continuously and causes enormous treatment costs. LPD with KA supplementation allows health care systems to save economic resources.

### Dose-response analysis regarding KA supplementation

To the best our knowledge, our study is the largest cohort study to date investigating the effectiveness of supplementing LPD with KA, and the first to conduct dose-response analysis regarding KA supplementation and outcome in patients with advanced predialysis CKD. Our findings reveal the importance of the dose-response effect regarding KA supplementation of LPD to improve the outcome. The association between the cumulative dose and the risk of long-term dialysis or the composite outcome in patients with advanced CKD deserves attention. This study suggests that supplementary KA of more than 5.5 tablets daily could reduce the risk of undesired outcomes; many of the enrollees in our cohort underuse KA, which has no beneficial effect on preserving kidney function. This suggests that underuse of KA may be a problem that calls for more attention in medical practice.

A recently published randomized, controlled trial by Garneata et al.[43] compared the safety and efficacy of ketoanalogue—supplemented vegetarian very low—protein diet (KD, 0.3 g/kg vegetable proteins per day) with conventional LPD (0.6 g/kg proteins per day) among patients with a stable estimated GFR<30 mL/min/1.73 m^2^. At 18 months of follow-up, compared with the LPD group, fewer patients in the KD group required RRT (30 versus 11 percent). The dose of ketoanalogues was 1 tablet per 5 kilogram body weight per day in that study. Noteworthy is the concern that Ketosteril with a maximum total dose of six tablets daily could yield a therapeutic effect or not, since six tablets daily is approximately equivalent to 1 tablet per 10 kilogram body weight per day, according to the maximum Taiwan NHI reimbursement regulations. Chen et al. conducted a prospective, group-comparison study[44] enrolled patients with serum creatinine more than 6 mg/dL. In their study, treatment with a low dose (Ketosteril one tablet/10 kg/day) of KA in CKD patients was not inferior to a standard dose (Ketosteril one tablet/5 kg/day) in preventing the progression of renal failure after 6-month observation. Further studies are required to confirm the dose-response relationship.

### The benefits of KA

Advanced CKD is not infrequently associated with protein-energy wasting and malnutrition[45]. KA has positive effects on protein metabolism, independent of the amount of dietary protein intake. Leucine as well as ketoleucine (a component of KA) plays a central role in promoting muscle protein synthesis by suppressing protein catabolism, with no change in the rate of protein synthesis [46, 47]. Oral supplementation of branched-chain amino acids can reduce anorexia sensation and significantly improves overall nutritional status in elderly malnourished hemodialysis patients [48]. In an observational study, malnourished advanced CKD patients treated with a KA supplement [46] had increased body weight, body mass index, serum albumin levels, and alleviated Subjective Global Assessment scores and appetite scores.

Hyperphosphatemia is linked to high all-cause mortality, adverse cardiac outcome, and rapid decline in renal function in CKD patients [49–51]. An LPD reduces phosphate intake, while KA is provided as calcium salts which reduces phosphate serum levels through formation of insoluble calcium phosphate in the intestines. Dietary protein restriction with supplemental KA was shown to significantly diminish proteinuria in CKD patients [52, 53]. Furthermore, regardless of drug class, for each 30% reduction in albuminuria the risk of ESRD decreased by 23.7%, and could further result in reduction of cardiovascular risks and heart failure [54]. KA supplemented LPD is associated with increased serum bicarbonate levels [6, 55], which in turn could improve nutritional status [56]. Long term co-administration of an LPD and KA in CKD patients decreased asymmetric dimethylarginine (ADMA) levels [38], thus attenuating endothelial dysfunction. This could slow kidney function progression and reduce all-cause mortality, and thus have a favorable effect on the composite outcome.

### Study strengths and limitations

A strength of our study is that the NHI database has a large national sample size and a long follow-up. Therefore, our analysis takes into account time-varying covariates and dose-response effect regarding KA supplementation, which can enrich the scope of future nutritional research in this field. However, our study has some limitations that should be acknowledged. First, the NHI research database does not contain information on self-pay use of medications and several potential confounders, including body mass index, smoking status, nutritional condition, data of proteinuria, serum bicarbonate, and the adequacy of glycemic, lipid, and blood pressure control. One study enrolled 3,320 advanced CKD patients in Taiwan showed that the overall mean BMI in male and female patients were 24.8 ± 3.4 kg/m^2^ and 24.3 ± 3.9 kg/m^2^, respectively [57]. Moreover, as in all observational studies using electronic databases, we were unable to confirm whether patients actually took the dispensed medications. Second, indications for dialysis and the decision of whether to receive dialysis is a complex issue that depends on several factors which are beyond the scope of this study. Generally, the clinical decision to start dialysis is taken late in Taiwan [14]. Although this makes no difference to survival compared with early dialysis, it provides the opportunity to observe the positive effect of supplemental KA. Third, this study design is a retrospective, observational cohort study; therefore it has inherent limitations in attributing improved outcomes to KA supplementation. Fourth, patients with acute kidney injury who had transient creatinine levels of more than 6 mg/dL were possibly included in this database. Therefore, we conducted an additional sensitivity analysis to restrict the analysis to patients receiving ESA therapy persistently at 2 or more consecutive ambulatory care visits, and the results were not materially changed. Furthermore, use of a serum creatinine level as a major inclusion criterion is also likely to lead to select the higher proportion of women than men in this cohort. However, in our subgroup analyses (Fig 3), KA supplementation was consistently associated with a much lower risk of long-term dialysis and composite outcome independent of age, sex and baseline comorbidities. Finally, the generalizability of our data is limited to the population of patients with advanced CKD who were receiving ESA therapy. These results may not be applicable to all patients with advanced CKD.

## Conclusions

Our study represents the largest national cohort study of long-term KA supplementation in advanced CKD patients. KA supplementation may substantially reduce the risk of commencing long-term dialysis or developing the composite outcome in advanced CKD patients on an LPD. Importantly, the favorable outcome of KA supplementation is evident only when its dosage is appropriate. Prescriptions corresponding to more than 5.5 tablets of KA daily are warranted. KA supplementation provides an additional therapeutic strategy to slow down the progression of CKD.

## Supporting information

S1 File **Table A**. Components of Ketosteril. **Table B**. Procedure and ATC codes. **Table C**. Comorbidity codes. **Table D**. Risk factors for chronic dialysis in advanced CKD patients receiving ESA treatment persistently. **Table E**. Risk factors for the composite outcome in advanced CKD patients receiving ESA treatment persistently. **Table F**. Risk factors for death among patients with advanced chronic kidney disease.
(DOC)
