# Supplementary material for: Ketoanalogues supplementation decreases dialysis and mortality risk in patients with anemic advanced chronic kidney disease
Source: PLoS One. 2017 May 5;12(5):e0176847. doi: 10.1371/journal.pone.0176847 (PMC5419544; doi:10.1371/journal.pone.0176847)
Supplement: S1 File — Table A. Components of Ketosteril. Table B. Procedure and ATC codes. Table C. Comorbidity codes. Table D. Risk factors for chronic dialysis in advanced CKD patients receiving ESA treatment persistently. Table E. Risk factors for the composite outcome in advanced CKD patients receiving ESA treatment persistently. Table F. Risk factors for death among patients with advanced chronic kidney disease. (DOC) [file pone.0176847.s001.doc]

**Ketoanalogues Supplementation Decreases Dialysis and Mortality Risk in Patients with Anemic Advanced Chronic Kidney Disease**

**Authors:** Che-Hsiung Wu^1, 2, 3^, Ya-Wen Yang^4^, Szu-Chun Hung^1^, Ko-Lin Kuo^1^, Kwan-Dun Wu^5^, Vin-Cent Wu^5^*^*^*, Tsung-Cheng Hsieh^2^*^*^,* the National Taiwan University Study Group on Acute Renal Failure (NSARF) ^#^

^1^ Division of Nephrology, Taipei Tzu Chi Hospital, Buddhist Tzu Chi Medical Foundation, Taipei, Taiwan

^2^ Institute of Medical Sciences, Tzu Chi University, Hualien, Taiwan

^3^ School of Medicine, Tzu Chi University, Hualien, Taiwan

^4^ Division of General Surgery, Department of Surgery, National Taiwan University Hospital, Taipei, Taiwan

^5^ Division of Nephrology, Department of Internal Medicine, National Taiwan University Hospital, Taipei, Taiwan

^#^ The full membership list of the National Taiwan University Study Group on Acute Renal Failure (NSARF) can be found in the Acknowledgements

*^*^* Correspondences and reprint requests to

**Vin-Cent Wu**, MD

Room 1419, Clinical Research Building, Department of Internal Medicine, National Taiwan University Hospital

7 Chung-Shan South Road, Taipei 100, Taiwan

Telephone: +886-2-23562082, Fax: +886-2-23934176, E-mail: [q91421028@ntu.edu.tw](mailto:q91421028@ntu.edu.tw)

And

**Tsung-Cheng Hsieh**

701, Chung Yang Rd., Sec.3, Hualien 970, Taiwan

Telephone: +886-3-8565301 #2015, E-mail: [tchsieh@mail.tcu.edu.tw](mailto:tchsieh@mail.tcu.edu.tw)

**Table A. Components of Ketosteril**

| **1 tablet of Ketosteril contains** | **(mg)** |
| --- | --- |
| Calcium-DL-3-methyl-2-oxovaleric acid  (α-Keto-analogue of DL-isoleucine, Clacium salt)  Calcium-4-methyl-2-oxovaleric acid  (α-Keto-analogue of Leucine, Calcium salt)  Calcium-2-oxo-3-phenylpropionic acid  (α-Keto-analogue of Phenylalanine, Calcium salt)  Calcium-3-methyl-2-oxobutyyric acid  (α-Keto-analogue of Valine, Calcium salt)  Calcium-DL-2-hydroxy-4-(methylthio)-butyric acid  (α-Keto-analogue of Methionine, Calcium salt)  L-lysine acetate  L-threonine  L-tryptophan  L-histidine  L-tyrosine | 67  101  68  86  59  105  53  23  38  30 |
| Total calcium  Total nitrogen | 50  36 |

**Table B. Procedure and ATC codes**

Dialysis

|  | **Procedure code** |  |
| --- | --- | --- |
|  | 58001C,58019C,58020C,58021C  ,58022C,58023C,58024C,58025C  ,58027C,58029C,58007C,58014C  ,58018C,58002C,58011A,58011B  ,58011C,58017A,58017B,58017C  ,58026C,58028C |  |

Drug

|  | **Procedure code** | **ATC CODE** |
| --- | --- | --- |
| EPO |  | B03XA |
| ACEI |  | C09AA |
| ARB |  | C09CA |
| Diuretic |  | C03AA, C03BA, C03CA |
| Ketosteril | BC18273100 |  |

**Table C. Comorbidity codes**

|  | **ICD9-codes** |
| --- | --- |
| **Myocardial infarction** | 410, 410.X, 410.XX, 412 |
| **Congestive heart failure** | 428,428.X |
| **Peripheral vascular disease** | 441, 441.X, 443.9, 785.4, V434 |
| **Cerebrovascular disease** | 430, 431, 432, 432.X, 433, 433.X, 434, 434.X, 435, 435.X, 436, 437,437.X, 438,438.X, 438.XX |
| **Dementia** | 290, 290.X, 290.XX |
| **Chronic pulmonary disease** | 490, 491, 491.X, 491.XX,492, 492.X, 493, 493.X, 493.XX,494, 495, 495.X, 496, 500, 501, 502, 503, 504, 505, 506.4 |
| **Reumatologic disease** | 710.X, 714, 714.X, 714.XX, 725 |
| **Peptic Ulcer** | 531, 531.X, 531.XX,532, 532.X, 532.XX,533, 533.X, 533.XX,534, 534.X, 534.XX |
| **Moderate or Severe liver disease** | 571.2, 571.4, 571.4X, 571.5, 571.6, 572, 572.X, 456, 456.0, 456.1, 456.2, 456.2X |
| **Diabetis Mellitus** | 250, 250.0, 250.0X, 250.1, 250.1X, 250.2, 250.2X, 250.3, 250.3X, 250.4, 250.4X, 250.5, 250.5X, 250.6, 250.6X, 250.7, 250.7X |
| **Hemiplegia** | 342, 342.X, 342.XX, 344.1 |
| **Tumor** | 140,140.X,141, 141.X,142, 142.X,143, 143.X,144, 144.X,145, 145.X,146,146.X,147,147.X,148,148.X,149,149.X,150,150.X  , 151, 151.X, 152, 152.X, 153, 153.X, 154, 154.X, 155, 155.X  , 156, 156.X, 157, 157.X, 158, 158.X, 159, 159.X,160,160.X, 161, 161.X, 162, 162.X, 163, 163.X, 164, 164.X, 165, 165.X, 166  , 166.X, 167, 167.X, 168, 168.X, 169, 169.X, 170,170.X,171,171.X,172,172.X, 174,174.X,175,175.X,176,176.X,179,180,180.X,181,182,182.X  ,183,183.X,184,184.X,185,186,186.X,187,187.X,188,188.X,189  ,189.X, 190,190.X,191,191.X,192,192.X,193,194,194.X,195  ,195.X, 200,200.X,200.XX,201,201.X, 201.XX,202,202.X,202.XX,  203,203.X,203.XX,204,204.X,204.XX,205,205.X,205.XX,206  ,206.X,206.XX,207,207.X,207.XX,208,208.X,208.XX |
|  | 196,196.X,197,197.X,198,198.X,198.XX,199,199.X, |
| **Hypertension** | 401, 401.X, 401.XX, 402, 402.X, 402.XX, 403, 403.X, 403.XX, 404, 404.X, 404.XX, 405, 405.X, 405.XX, |
| **Stroke** | 43X, 43X.X |
| **Nephrotic syndrome** | 581.X, 581.XX |
|  |  |

**Table D. Risk factors for chronic dialysis in advanced CKD patients receiving ESA treatment persistently**

| **Variables** |  | **Hazard Ratio ( 95% confidence interval)** | **p value** |
| --- | --- | --- | --- |
| **Diabetes mellitus** | | 1.86 ( 1.62 – 2.13 ) | < 0.001 |
| **Hypertension** | | 1.35 ( 1.19 - 1.53 ) | < 0.001 |
| **Age, >60 vs. ≦60 years old** | | 1.19 ( 1.05 - 1.34 ) | 0.0052 |
| **Daily KA dosage ( expressed as tablets)** ^b^ **larger than 5.5 vs. Unsuitable dose** | | 0.63 ( 0.41 - 0.97 ) | 0.0358 |
| **KA use** | | 0.52 ( 0.45 - 0.59 ) | < 0.001 |
| **ARB use** | | 0.76 ( 0.65 - 0.89 ) | < 0.001 |

^b^KA prescribed at less than 5.5 tablets daily is considered an unsuitable dose

Factors used in the Cox model: all covariates listed in Table 1

**Abbreviations:** ARB, angiotensin II receptor antagonist; KA, Ketoanalog

**Table E. Risk factors for the composite outcome in advanced CKD patients receiving ESA treatment persistently**

| **Variables** |  | **Hazard Ratio ( 95% confidence interval)** | **p value** |
| --- | --- | --- | --- |
| **Diabetes mellitus** | | 1.53 ( 1.34 - 1.75 ) | <0.001 |
| **Hypertension** | | 1.27 ( 1.13 - 1.43 ) | <0.001 |
| **Diuretic use** | | 2.11 ( 1.88 - 2.42 ) | <0.001 |
| **KA use** | | 0.43 ( 0.37 - 0.49 ) | <0.001 |
| **Daily KA dosage ( expressed as tablets)** ^b^  **larger than 5.5 vs. Unsuitable dose** | | 0.61 ( 0.40 - 0.93 ) | 0.0208 |

^b^KA prescribed at less than 5.5 tablets daily is considered an unsuitable dose

Factors used in the Cox model: all covariates listed in Table 1

**Abbreviations:** KA, Ketoanalog

**Table F. Risk factors for death among patients with advanced chronic kidney disease**

| **Variables** |  | **Hazard Ratio ( 95% confidence interval)** | **p value** |
| --- | --- | --- | --- |
| **Diabetes mellitus** | | 1.51 ( 1.18 – 1.93 ) | 0.0011 |
| **Hypertension** | | 1.31 ( 1.02 - 1.66 ) | 0.0308 |
| **Age, >60 vs. ≦60 years old** | | 1.07 ( 1.06 - 1.08 ) | < 0.001 |
| **Congestive heart failure** | | 1.61 ( 1.04 – 2.48 ) | 0.0314 |
| **Male** | | 1.35 ( 1.08 – 1.69 ) | 0.0077 |
| **KA use** | | 0.26 ( 0.16 - 0.43 ) | < 0.001 |
| **Diuretics use** | | 2.15 ( 1.63 – 2.84 ) | < 0.001 |

Factors used in the Cox model: all covariates listed in Table 1

**Abbreviations:** KA, Ketoanalog
